# *In vitro* and *ex-vivo* cellular antioxidant protection and cognitive enhancing effects of an extract of *Polygonum minus* Huds (Lineminus^™^) demonstrated in a Barnes Maze animal model for memory and learning

**DOI:** 10.1186/1472-6882-14-161

**Published:** 2014-05-19

**Authors:** Annie George, Chee Perng Ng, Matthew O’Callaghan, Gitte S Jensen, Hoi Jin Wong

**Affiliations:** 1 Biotropics Malaysia Berhad, Lot 21, Jalan U1/19, Section U1, Hicom-Glenmarie Industrial Park, Shah Alam, Selangor 40150, Malaysia; 2 Cerca Insights Sdn Bhd (NCIA Technology Development Center) Level 2, Plot No.36 Hilir Sungai Keluang, Bayan Lepas Industrial EstatePhase IV, Bayan Lepas, Penang 11900, Malaysia; 3 NIS Labs, 1437 Esplanade, Klamath Falls, OR 97601, USA

**Keywords:** Scopolamine, Antioxidant, Barnes maze, *Polygonum minus*, Cognition

## Abstract

**Background:**

*Polygonum minus* Huds.is a culinary flavouring that is common in South East Asian cuisine and as a remedy for diverse maladies ranging from indigestion to poor eyesight. The leaves of this herb have been reported to be high in antioxidants. Flavonoids which have been associated with memory, cognition and protection against neurodegeneration were found in *P. minus*.

**Method:**

This study examined a *P. minus* aqueous extract (Lineminus^™^) for its antioxidant activity using the Oxygen Radical Absorbance Capacity (ORAC) assay, the *ex vivo* Cellular Antioxidant Protection of erythrocytes (CAP-e) assays and for potential anticholinesterase activity *in vitro*. Cognitive function and learning of Lineminus^™^ was evaluated using scopolamine induced cognition deficits in a Barnes maze, rodent model of cognition.

**Results:**

The extract displayed *in vitro* antioxidant activity with a total ORAC value of 16,964 μmole TE/gram. Cellular antioxidant protection from free radical damage using the CAP-e assay, with an IC50 of 0.58 g/L for inhibition of cellular oxidative damage, was observed. The extract inhibited cholinesterase activity with an IC50 of 0.04 mg/ml with a maximum inhibition of 68%. In a rodent model of cognition using scopolamine induced cognition deficits in the Barnes maze, the extract attenuated scopolamine induced disruptions in learning at the higher dose of 100 mg/kg.

**Conclusion:**

These data shows that *P. minus* possesses antioxidant and anticholinesterase activity and demonstrated enhanced cognition *in vivo*. The data suggest neuroprotective properties of the extract.

## Background

*Polygonum minus* Huds. synonymous to *Persicaria minor* is from the family Polygonaceae and is commonly referred to as Kesum or laksa leaf in Malaysia. It is used as a flavouring ingredient in culinary dishes and also consumed as an ulam (salad) for preventive healthcare [1,2]. Traditionally, the decoction of leaves is taken for indigestion, after childbirth, to warm the body up and to promote good eyesight [3]. It is possible that *P. minus* has the ability to increase blood circulation [4].

Several studies have shown that leaves of *P. minus* are high in antioxidants [5-9]. It has been reported that water extracts of *P. minus* have shown superior antioxidant activity when compared to other popular herbs such as ulam raja, selom, pegaga and curry leaves, where antioxidant activity was similar to the synthetic antioxidant butylhydroxytoluene (BHT) [6]. Water extracts of *P. minus* had higher antioxidant activity, measured by the total phenolic content (TPC), when comparing to ginger (*Zingiber officinale*) and turmeric (*Curcuma longa*) [9], 2,2-diphenyl-11-picryhydrazyl free radical (DPPH) and ferric reducing antioxidant power (FRAP) [7]. *Polygonum minus* was shown to possess 98.3% of lipid peroxidation inhibitory activities and is proposed as a candidate for nutraceutical and cosmeceutical product [2]. It has also been reported to be rich in vitamins such as carotenes, retinol equivalents and vitamin C, α-tocopherol (vitamin E) and minerals such as calcium, phosphorus, iron, sodium, potassium, magnesium, copper and zinc [10].

Free radicals, including reactive oxygen species, have been shown to cause aging and several degenerative diseases such as atherogenesis, cardiovascular and neurodegenerative diseases [11,12]. Although the production of free radicals is a normal by-product of metabolism and environmental stress, the over production leads to cell damage. For this reason, antioxidants such as vitamins A, C, E, carotenoids and plant polyphenols (flavonoids, phenolic acids, cathechins, coumarins, tannins and anthocyanins) are commonly consumed as part of the food composition as a protective defence mechanism against such damage. Plant antioxidants can therefore act as agents in scavenging reactive oxygen species.

Phytochemical screening of *P. minus* have shown the presence of flavanoids, flavones, cathecin, epicatechingallate and terpenoids [5]. Flavonoid and polyphenols have been long studied for their strong antioxidant capacities,and their ability to scavenge reactive oxygen species thus preventing aging and oxidative stress related diseases [13,14]. Studies also show that flavonoids have an effect on memory, cognition and against neurodenegeration and the ability to improve cerebrovascular blood flow [15].

The nutraceutical industry uses a standardized chemical antioxidant method, ie. the Oxygen Radical Absorbance Capacity (ORAC) test to evaluate antioxidant strength. The ORAC is a HAT-based assays measuring the capability of an antioxidant to quench free radicals (generally, peroxyl radicals) by H-atom donation [16]. In addition, the ORAC assay measures the degree and length of time the extracts take to inhibit the action of an oxidizing agent unlike the DPPH assay. The ORAC was identified as one of the best standardized antioxidant assay for the natural products industry in measuring antioxidant levels in nutritional and natural products [17-19]. The U.S. Department of Agriculture developed a database of biological materials and foods to provide the basis for comparing antioxidant strength based on ORAC values [20]. This enables one to compare the antioxidant levels against other popularly known antioxidant foods hence making it easier to compare the antioxidant effect of *P. minus* as a food with other more established high antioxidant foods. The ORAC assay has since been commercialized by Brunswick Labs, Wareham, MA, USA. The DPPH and FRAP method on the other hand is a SET method, which measures the ability of the antioxidant to transfer one electron to reduce a specific oxidant. In the case of DPPH assay, it measures direct reactions with the DPPH radical, which is dependent on the structure of an antioxidant compound therefore only giving a general indication of the radical scavenging abilities of antioxidants.While DPPH method is a rapid and convenient method to measure antioxidant activity it diverges from biological conditions the most, by the use of an artificial DPPH radical and methanol as the solvent [21]. This is in contrast to ORAC which is performed at a physiological pH producing a biologically relevant radical, the peroxyl radical [22].

However, there is a need to demostrate the antioxidant capacity in serum to show bioavailibity. Since a clinical trial is costly and time consuming, a more intermediary cell-based study was developed to test to what extent a substance protects against oxidative stress in a biologically relevant sytem. The Cellular Antioxidant Protection of erythrocytes (CAP-e) assay is a cell-based antioxidant protection assay using erythrocytes to address the question of whether antioxidants in complex natural products enter the cytosol and contribute to the reduction of oxidative damage within the cell [23] and was successfully applied in other antioxidant rich herb and fruit such as the Acai berry [24]. The current study is an attempt to draw a parallel between the antioxidant property of the herb, its’ relevance in a biological system, using the CAP-e assay (ex vivo) and the improvement of learning and memory *in vivo* as one of the manifestation of the antioxidant property.

As improvements in cogniton may be multi-pronged, the herb is also tested for antiacetylcholinesterase activity. Acetylcholine is a neurotransmitter related to learning and memory [25]. It is metabolised by the enzyme acetylcholinesterase. Inhibition of acetylcholinesterase is presently the most accepted and recognized therapeutic marker for the development of cognitive enhancers [26]. Anti-cholinesterase activity has never been tested for *P. minus.* Screening for herbal plants with acetylcholinesterase inhibitory activity would open new possibilities for cognition improving herbal products.

Several *in vivo* models have been used to investigate learning and memory in animal models of which Scopolamine, a muscarinic receptor antagonist, produce deficits in spatial navigation tasks in rodents [27]. Scopolamine significantly increases acetylcholinesterase (AChE) and malondialdehyde (MDA) levels in the cortex and hippocampus [26].

The Barnes maze was developed as a sensitive tool for testing hippocampus-dependent spatial memory in rats [28] and is the model adopted for this study. In addition, for mice the Barnes maze is better, as they swim less well than rats. The Barnes maze is similar to the Morris water maze task, but does not utilize a strong aversive stimulus (stress induced by swimming as reinforcement). Behavioral tasks involving high levels of stress can influence the animal's performance [29].

This study was performed to investigate *P. minus* in protecting against oxidative stress in a cell-based study and in memory improvement *in vivo.* Currently the plant extract most popularly researched for the ability to enhance cognition is *Gingko biloba*. Extracts of *G. biloba* were shown to improve memory and normalizing cognitive deficits in animal models [30], and in treatment of cognitive improvement in Alzheimer’s patients [31]. In this study, the antioxidant activity of *P. minus* was tested *in vitro* and the protection against oxidative damage demonstrated in red blood cells. The paper attempts to draw a parallel between the protective antioxidant affect of the herbal extract to the cognition enhancing effect, in an animal model induced with cognitive deficits by scopolamine, whose activity can also be attenuated by an anti acetylcholinesterase. The activity of the herb was compared to the more traditionally and scientifically documented, *G. biloba* which has been reported to possess anti-acetylcholinesterase activity and improve cognition *in vivo*.

## Methods

### Plant material

*Polygonum minus* was procured from Biotropics Malaysia Berhad, Malaysia. The plant material was identified based by a Taxonomist from Institute Bio Science, University Putra Malaysia (UPM) based on their exomorphic characters and literature review of the plant. The voucher specimen of the plant (SK 2077/12) was deposited in the Herbarium Institute Bioscience, UPM Malaysia. The aerial parts of plants including stem and leaves were extracted to produce an aqueous extract for *in vitro* and *in vivo* assays.

### Plant extract

**Figure 1:**
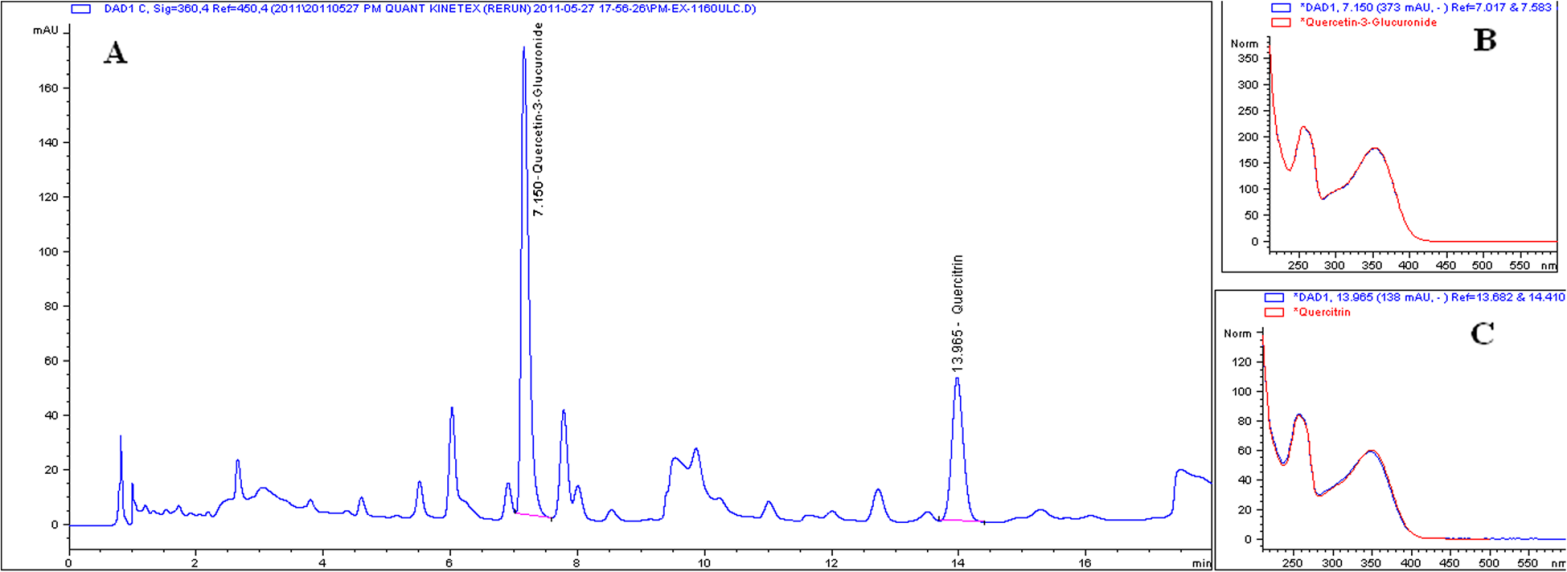
**HPLC profile of *Polygonum minus *water extract. A**. The peaks correspond to 2 compounds identified as Quercetin-3-glucuronide and Quercitrin identified at RT 7.150 min and 13.975 min, respectively. **B**. The UV spectrum of Quercetin-3-glucuronide identified with respect to its’ retention time. **C**. The UV spectrum of Quercitrin identified with respect to its’ retention time.

Fresh plant material was oven-dried to below 10% moisture content. The dried leaves were chopped into fragments and the extraction was performed by immersing these leaves in water at a ratio of 1:20 and percolated for 2 cycles for 4 hours at 80°C. The liquid was then filtered and evaporated. The liquid concentrate was subsequently freeze dried until it reached a moisture content of below 8% w/w. The extract was then vacuum packed in aluminum foil to preserve it in a cool low humidity with no direct exposure to sunlight. The water extract of *P. minus*, standardised to Quercetin-3-glucuronide 0.59% and 0.27% Quercitrin was prepared by Biotropics Malaysia Berhad according to process outlined in Malaysian Patent Pending No. PI2012003882 [32]. The HPLC fingerprint (Figure 1A) of *P. minus* water extract was obtained according to the HPLC method using Kinetex 1.7 μm C18 (2.1 × 150 mm) column. The mobile phase consisted of solvent A-0.10% formic acid in water and B-0.10% formic acid in acetonitrile mixed according to a linear gradient program of between 5-89% of solvent A and 95-11% of solvent B. Two major peaks in the fingerprint profile were isolated and identified to be quercetin-3-glucuronide and quercitrin based on their mass(es) and MS fragmentations. LC-MS-MS was performed using a Shidmadzu UFLC system equipped with a PDA and IT-TOFMS. Peaks at retention times 7.15 and 13.96 min identified as Quercetin glucuronide and Quercitrin respectively were further confirmed by comparing their retention time values and the obtained UV max with those of the standards. (Figure 1A-C).

The comparative plant extract of *Gingko biloba* was based on commercially available standardised extract of dried leaf from Shanghai Novanat Co. Ltd. The extract was standardised to 27.25% Gingkoflavoglycosides, 6% Terpene lactones and ≤ 5 ppm Ginkgolic acid determined through HPLC methods and passed microbial and heavy metal test (based on the Certificate of Analysis).

### Determination of antioxidant capacity using ORAC assay

Extract of *P. minus* was shipped to Brunswick Laboratories, Norton, MA, an independent contract laboratory specialising in standardised natural product assays, to test for ORAC values. Data were obtained for ORAC hydrophilic testing using fluorescein as the fluorescent probe and 2,2′-azobis (2-amidinopropane) dihydrochloride as a peroxyl radical generator, ORAC lipophilic testing for lipid antioxidants capable of quenching peroxyl free radicals, HORAC testing for antioxidants capable of quenching hydroxyl free radicals, NORAC testing for antioxidants capable of quenching peroxynitrite, and SORAC testing for superoxide dismutase-like activity (based on Certificate of Analysis released by the lab) [33].

### Determination of CAP-e antioxidant capacity

The CAP-e antioxidant capacity was estimated according to the modified method of Honzel [23], modified for a more sensitive and accelerated protocol [33]. An amount of 0.5 g of plant extract was mixed with 5 mL 0.9% saline at physiological pH, mixed by inversion, vortexed and allowed to incubate on a rocker for 20 minutes. The solids were removed by centrifugation at 2400 rpm for 10 minutes. The supernatant was removed and then filtered through a 0.22 micron cellulose acetate syringe filter before use in the CAP-e assay. Serial dilutions were prepared from the filtered supernatant in 0.9% saline at physiological pH. Red blood cells were treated in duplicate with serial dilutions of the test product. Samples of untreated red blood cells (negative controls) and samples of red blood cells treated with oxidizing agent but not with an antioxidant-containing test product (positive controls) were prepared in hexaplicate. The antioxidants not able to enter the cells were removed by centrifugation and aspiration of supernatant above the cell pellet.

The cells were exposed to oxidative damage by addition of the peroxyl free-radical generator 2,2’ azobis (2-amidinopropane hydrochloride) (AAPH). Using the indicator dye Dichlorofluorescein diacetate (DCF-DA), which becomes fluorescent as a result of oxidative damage, the degree of antioxidant damage was recorded by measuring the fluorescence intensity of each sample in a TECAN Spectrafluor plate reader. The inhibition of oxidative damage was calculated as the reduced fluorescence intensity of product-treated cells, compared to cells treated only with the oxidizing agent, in reference to the baseline levels of oxidation in untreated cells. The CAP-e value which is in Gallic Acid Equivalent (GAE) units, reflects the IC50 dose of the test products, i.e. the dose that provided 50% inhibition of oxidative damage. This is then compared to the IC50 dose of the known antioxidant Gallic acid.

### Determination of anticholinesterase activity

The extracts was screened for anticholinesterase activity using ProfilingScreen procured from Ricerca Pharmacology Services, Taiwan. The extract was tested in duplicates at a concentration of 10, 30 and 100 μg/ml. Concurrent vehicle 1% DMSO and reference standard Physostigmine were conducted with each assay using Human Recombinant HEK-293 cells.

### Animals

Two to six month old adult male C57BL/6 mice (20–25 g), (n = 12–14) were supplied by BioLASCO (Taiwan). The mice were group-housed under a 12/12-h light/dark (200–300 Lux) cycle (lights on 07:00 h) with free access to food (Labdiet, formulated laboratory chow) and water and humidity kept between 50%–70%. The experiment was approved by the Institutional Animal Care and Use Committee of Cerca Insights Sdn Bhd and was conducted in accordance with the Singapore NACLAR Guide (2004) for the care and use of laboratory animals.

### Treament

Herbal extract treatments of 50 mg/kg *P. minus*, 100 mg/kg *P.minus,* 50 mg/kg *G.biloba* or vehicle (water) were given daily via oral gavage for fourteen days prior to Barnes maze testing. This treatment continued during the five days of Barnes maze testing. During the Barnes maze testing these mice received i.p. injections of either scopolamine (0.5 mg/kg) or saline vehicle. A further group received i.p. injections of donepezil (1 mg/kg) and scopolamine (0.5 mg/kg). The dose and time of scopolamine administration has been previously shown to produce deficits in spatial navigation tasks in rodents [27].

### Barnes assay

The Barnes maze (BM) was created to evaluate spatial learning [18,26]. The Barnes maze consisted of a PVC circular platform with 21 holes placed 6 cm from the edge and equally distributed around the surface. The platform was 122 cm in diameter and 92 cm from the ground. The maze uses rodents natural aversion to open illuminated places and so the subjects were motivated by bright light (Lux level 300 – 500) to locate an escape hole which leads to a dark box (5.4 × 23 × 4.5 cm). Room design and equipment around the maze were used as fixed spatial cues (extra-maze cues) for navigational purposes.

Barnes maze testing consisted of three phases, an adaptation period, an acquisition period and a probe trial. A pre-trial (adaptation period) was given prior the start of trial on days one and two. Each subject underwent four trials per day for four days (acquisition period) and then a probe trial was performed twenty-four hours after the final acquisition trial. Thirty minutes prior to the first trial the test subjects received an injection of either scopolamine, saline vehicle, or scopolamine and donepezil.

### Adaptation period

The subject was placed in the center zone of the maze, shrouded in a chamber for 10 seconds. The chamber was removed and the subject was allowed to explore the maze for 30 seconds, then gently guided to the escape hole. If the subject did not then enter the escape hole, it was placed inside. The hole was then covered and the subject remained there for 3 minutes. The subject was then returned to its home cage and the platform cleaned with 70% ethanol.

### Acquisition period

The chamber was removed and the subject was allowed to explore the maze for 5 minutes and then gently guided to the escape hole. If the subject did not then enter the escape hole, it was placed inside. The hole was then covered and the subject remained there for 1 minute. The subject was then returned to its home cage and the platform cleaned with 70% ethanol. The next trial was run after following an inter-trial interval of two minutes.

### Probe trial

For the probe trial, an identical white disc was placed on the platform that covered all the holes. The subject was placed in the center zone of the maze, shrouded in a chamber for 10 sec. The chamber was removed and the subject was allowed to explore the area for 90 seconds. The subject was then returned to its home cage and the platform cleaned with 70% ethanol.

The behavior of the experimental subjects was captured by video camera and recorded on the hard drive of a desktop PC. An analysis of these recordings was performed using EthoVision® XT tracking system for the automatic tracking and analysis of animal movement. For each parameter, the performance in the trials on each day were averaged. The following parameters were measured during the test and processed - (i) Total path length, the distance moved by the subject during the entire session. Total path length is the total distance moved over the whole time of the experimental trial including the distance moved after the first encounter with the escape-hole (when the mouse has encountered the escape hole but failed to enter it). An increase in path length demonstrates a decrease in performance; (ii) Total errors, the count of the number of errors made by the subject throughout the trial. Total errors are the number of approaches to the non-escape hole, when the mouse has interacted with the escape hole but not escaped and further explored the maze. Both total path length and total errors measure learning; (iii) Total latency, the latency for subject to complete the task. Total latency describes the time taken by the mouse to enter the escape hole; (iv) For the probe trial the arena was divided into 8 right equal segments and duration in each segment by the subject was measured. The mice are allowed to explore the maze and the time the mice stay in various areas on the maze is recorded. This ‘probe’ trial is used to assess memory.

Data was analyzed using two-way repeated measures ANOVA, with ‘day’ as repeated-measure factor within subjects and ‘treatment group’ as between-subject’s factor. Post-hoc pairwise comparisons between groups using Tukey HSD test were carried out if significant effect was found. Data analysis was performed using Sigma Plot statistical software.

## Results

### Antioxidant capacity as measured by the ORAC assay and anti-cholinesterase activity

There are five predominant reactive species found in the body: peroxyl radicals, hydroxyl radicals, peroxynitrite, super oxide anion and singlet oxygen. Total ORAC_FN_ provides a measure of the total antioxidant power of a food/nutrition product against the five predominant reactive species. The ORAC_FN_ values for the extract are shown in Table 1.

**Table 1:** **Total ORAC**_**FN **_**showing a measure of the total antioxidant power of *P.minus *** **aqueous extract against the five predominant reactive species**

	
Antioxidant power against peroxyl radicals	2,591 μmole TE/gram
Antioxidant power against hydroxyl radicals	8,973 μmole TE/gram
Antioxidant power against peroxynitrite	222 μmole TE/gram
Antioxidant power against super oxide anion	4,039 μmole TE/gram
Antioxidant power against singlet oxygen	1,139 μmole TE/gram
Total ORAC_FN_ (sum of above)*	16,964 μmole TE/gram

*Brunswick Laboratories, USA [34].

The extract inhibited cholinesterase activity with an IC50 of 0.0405 mg/ml with maximum inhibition of 68%.

### CAP-e assay

**Figure 2:**
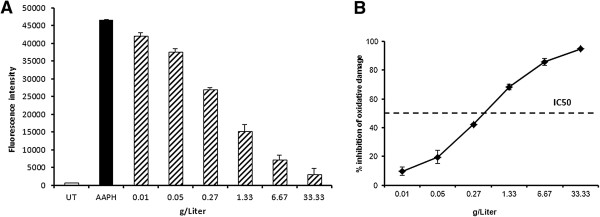
**A-B Cellular antioxidant protection of erythrocytes (CAP-e) by *P. minus*. A**. Erythrocytes were either tested for baseline levels of oxidation (untreated cells, UT), or under conditions of oxidative stress (AAPH, black bar). In parallel, erythrocyte cells were exposed to serial dilutions of *Polygonum minus* before exposure to oxidative stress (dashed bars). The pre-treatment of cells before exposed to oxidative stress resulted in significant cellular antioxidant protection, shown as the dose-dependent reduction in oxidation (measured by fluorescence intensity (FI) of the cells). **B**. The dose-dependent inhibition of cellular oxidative damage as a result of *Polygonum minus*treatment is shown, calculated from fluorescence intensity (FI) of samples and controls: (FI(max) – FI(sample)/FI(max)-FI(UT)).

The CAP-e assay was used to test whether the extract contained antioxidants capable of protecting cells from oxidative damage. The inhibition of oxidative damage was calculated as the reduced fluorescence intensity of product-treated cells, compared to cells treated only with the oxidizing agent in the absence of antioxidant protection. The CAP-e value reflects the IC50 dose of the extract, i.e. the dose that provided 50% inhibition of oxidative damage. The CAP-e value for the *P. minus* extract was shown to be 55 gallic acid equivalents per gram extract, based on an IC50 value of 0.58 g/L (Figure 2A-B).

### Learning phase (acquisition training)

**Figure 3:**
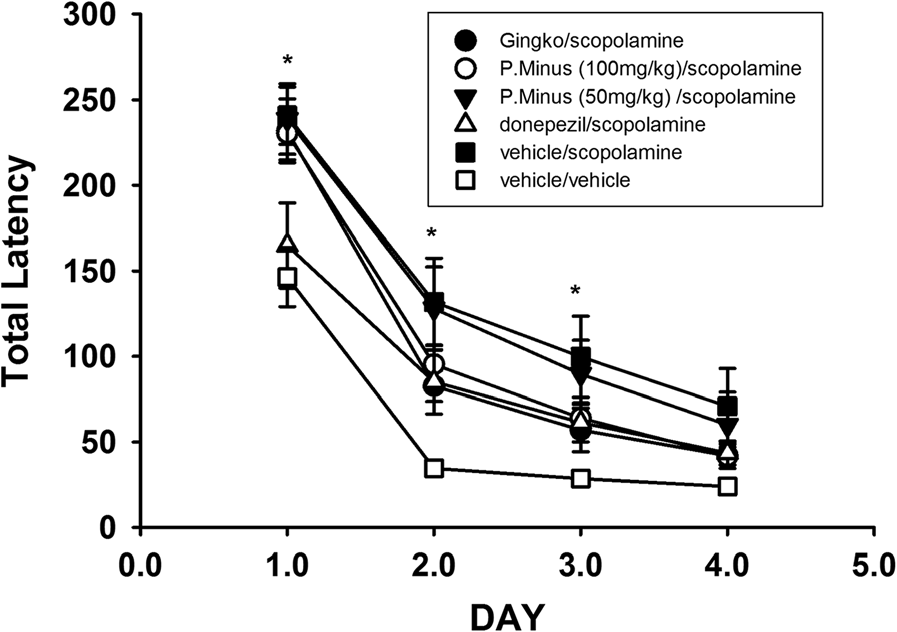
**Total latency in the time taken to escape.** Scopolamine treated mice showed a significant increase in latency when compared to vehicle treated mice (*P > 0.05). Total latency decreases for all treatments over time.

A two way repeated measures ANOVA of total path length indicated a significant effect of both treatment (F_5,72_ = 4.4, p < 0.01) and day (F_3,70_ = 65.99,p < 0.001) as shown in Table 2. The days one, two, three or four on which the animal was tested is a significant factor in performance. Post-hoc analysis showed a significant increase in path length in the scopolamine treated control mice on days one, two and three (p < 0.05) compared to vehicle treated mice indicating a scopolamine induced deficit in learning. Hence, an increase in path length demonstrates a decrease in performance. This increase in path length was not seen in the mice treated with *G. biloba*, donepezil or 100 mg/kg *P. minus*. The scopolamine induced deficit was not reversed by treatment with 50 mg/kg *P. minus*. These mice showed a significant decrease in performance when compared to vehicle treated mice on day two and three. However, the donepezil, *G. biloba* and the 100 mg/kg *P. minus* treated mice did not show a scopolamine induced deficit. There was a significant effect of day (F_3,70_ = 62.56, p < 0.0001) and treatment (F_5,72_ = 6.13, p < 0.001) on total errors. A significant increase in total errors was seen in the scopolamine treated mice on days one and two (p < 0.05). A significant increase in total errors was also seen in *P. minus* (50 mg/kg) treated mice on days two and three (p < 0.05). The deficits for total errors were not observed after treatment with *P. minus* at 100 mg/kg, *G. biloba* or donepezil for days one, two and three (Table 2). There was a significant effect of day (F_3,70_ = 111.1,p < 0.0001) and treatment (F_5,72_ = 4.94, p < 0.001) on total latency. A significant increase (p < 0.05) in total latency was see in the scopolamine and *P. minus* (50 mg/kg) treated mice on days one, two and three (Figure 3).

**Table 2:** Total path length travelled in the Barnes maze and total errors

	**Total path length (Mean ± SE)**				**Total errors (Mean ± SE)**			
**Treatment**	**Day-1**	**Day-2**	**Day-3**	**Day-4**	**Day-1**	**Day-2**	**Day-3**	**Day-4**
***G. biloba *** **50 mg/kg**	1538.99	497.76	324.95	212.4	32.35	11.9	9.46	8.00
	±185.09	±73.89	±82.76	±25.16	±3.54	±1.69	±2.77	±1.34
***P. minus *** **50 mg/kg**	1420.44	732.61	556.98±	340.38	33.77	22.03	18.77	12.18
	±171.2	±137.06^a^	117.97^a^	±65.71	±3.67	±4.25^a^	±3.83^a^	±2.41
***P. minus *** **100 mg/kg**	1436.37±	602.87	356.95±	244.47	31.92	15.71	10.85	7.88
	160.44^b^	±96.82	51.28	±26.89	±3.06	±2.2	±1.78	±1.18
**Donepezil 1 mg/kg**	1031.39	559.37±	396.59±	305.24	20.79	12.95	11.36	9.00
	±173.06^b^	137.29	71.57	±36.59	±3.65	±2.5	±1.6	±1.09
**Veh/Sco**	1841.41±	817.31 ± 1	537.84±	365.66	44.06	21.65	16.58	13.00
	238.12^a^	90.27^a^	111.44^a^	±86.44	±5.22^a^	±3.91^a^	±3.27	±2.74
**Veh/Veh**	926.06 ± 1	234.06 ± 2	202.36±	211.82	19.09	6.75	5.98	6.40
	96.13^b^	7.8^b^	24.26	±30.61	±3.19	±1.24	±0.87	±1.27

^a^Significance when compared to control vehicle + vehicle, p < 0.05.

^b^Significance when compared to control vehicle + scopolamine, p < 0.05.

Veh = vehicle; Sco-Scopolamine; Don = Donepezil.

### Memory phase (probe trial)

One-way ANOVA demonstrated significant duration i.e. time spent in the target segment in the vehicle treated control (F_7,103_ = 6.86, p < 0.0001); the scopolamine with donepezil treated (F_7,111_ = 6.94, p < 0.0001); the *G. biloba* treated (F_7,100_ = 4.2333, p < 0.001) and the *P. minus -*100 mg/kg (F_7,95_ = 6.6852, p = 0.01) mice, but not the scopolamine alone nor the scopolamine plus *P. minus*- 50 mg/kg treated mice (Table 3). This suggests that scopolamine has induced a deficit in memory which was not reversed by the low dose of *P. minus.* Both *G. biloba*, donepezil and higher dose of 100 mg/kg *P.minus* reversed scopolamine induced memory deficits.

**Table 3:** Duration in target segment during probe trial

**Duration in target segment during probe trial (sec)**					
**Day**	**Treatment**	**Mean**	**SE**	**F value**	**P value**
**5**	***G.biloba *** **50 mg/kg**	20.5938462	3.44090282	(7,100) = 4.2333	p = 0.0004***
**5**	***P.minus *** **50 mg/kg**	18.3257143	3.70012143	(7,105) = 1.7621	p = 0.1036 n.s
**5**	***P.minus *** **100 mg/kg**	19.7230769	2.53861445	(7,95) = 6.6852	p = 0.0015**
**5**	**Donepezil 1 mg/kg**	16.6314286	1.65977983	(7,111) = 6.9417	p < 0.0001***
**5**	**Veh/Sco**	16.5533333	2.48504781	(7,88) = 1.7926	p = 0.0999 n.s
**5**	**Veh/Veh**	19.7384615	1.69910151	(7,103) = 6.8633	p < 0.0001***

***, One-way ANOVA demonstrated significant duration in the target segment p < 0.0001.

**, One-way ANOVA demonstrated significant duration in the target segment p < 0.001.

n.s, non significant.

## Discussion

The extract was shown to possess strong antioxidant capacity, as measured by the oxygen radical absorbance (ORAC assay). The ORAC value of *P. minus* water extract can now be compared to the more popularly known high antioxidant foods such as Granny Smith apples, cranberry and blueberry at ORAC values of 5381, 8983 and 9019 μmol TE/100 g respectively [20]. In addition, the antioxidants of the standardised extract are capable of entering into and protecting cells from oxidative damage, as shown by the cellular antioxidant protection (CAP-e) assay. These properties may contribute to the effects seen in the animal model of cognitive function, since the brain has a high level of metabolism and oxygen use and so is susceptible to oxidative attack by free radicals. Additionally, it has a relatively low concentration of anti-oxidative enzymes and free radical scavengers [35]. Previous reports have suggested that a water extract of *P. minus* promotes high antioxidant levels determined by free radical scavenging activity of DPPH radical and the ability of antioxidant in this plants extract to reduce ferric (III) iron to ferrous (II) iron in FRAP reagent, probably as a result of high flavonoid and total phenolic content [6,9]. Using different methods to measure antioxidant property, the antioxidant property for water extracts of *P. minus* was demonstrated and by evaluating ORAC values, the standardised *P. minus* extract-Lineminus™ could be compared with other antioxidant foods.

In this study, we managed to evaluate antioxidant effect *ex-vivo* by the use of erythrocyte cells (in the CAP-e bioassay study), where oxidative stress was reduced in a dose dependent manner by the presence of the extract. Natural products including flavonoids that exhibit anti-oxidative effects have been found to attenuate memory impairments [24]. Some isolated antioxidant compounds, including luteolin, quercetin, and dihydrokaempferol, from Acai fruit pulp had the capacity to enter live cells and protect them from oxidative damage, demonstrated by using the same Cellular Antioxidant Protection in erythrocytes (CAP-e) bioassay [24]. Some flavonoids in fact have been reported to cross blood brain barrier *in vitro*[36]. The *in vivo* studies showed that flavonoids are able to be absorbed after oral administration, pass the blood–brain barrier and do have various effects on the CNS [37]. Derivatives of quercetin and flavonoids were identified in extracts of *P. minus*[8], possibly leading to the CAP-e effect observed in this study. However, the concentrations of flavonoids and their metabolites which reach the brain in the current study have to be assessed. If this antioxidant activity is confirmed *in vivo*, this could help reduce increased oxidative stress such as that reported to occur in the aging brain and so may be therapeutically useful. Hence the possibility of flavonoids of *P. minus* to cross blood brain barrier thus qualifying the procognitive effect in the animal study conducted here, should be further investigated.

In the present study, scopolamine treatment induced deficits during the early part of the acquisition (learning) phase of the Barnes maze task. The scopolamine induced deficits in learning were evidenced by an increase in the number of errors, latency, and path length. Considering the learning curve, the impairment of scopolamine treated animals seemed most pronounced early in training, after which some constancy was reached between the treatment groups. This is best illustrated by the increased path length in the scopolamine treated mice on days one, two and three, but not day four. These deficits were attenuated by the positive controls – donepezil and the *G. biloba* extract.They have both been reported to reverse scopolamine induced deficits in learning [29]. The *P. minus* extract, when administered at 100 mg/kg, attenuated scopolamine induced deficits in the acquisition phase of the Barnes maze task.

The dose of 100 mg/kg *P. minus* extract also reversed scopolamine induced deficits in the retention (probe trial) aspect of the task. In the probe trial, all the treatments except the lower dose of 50 mg/kg of *P. minus* attenuated scopolamine induced deficits. These deficits were described by no significant preference for the target segment by the mice. The lack of effect of the lower dose and the significant effect of the higher dose would suggest that there was a dose dependent action of the extract. The results suggest that an extract of *P. minus* can attenuate scopolamine induced learning and memory deficits in mice.

Decreases in cholinergic tone are associated with cognitive dysfunction and are reported in neurodegenerative diseases such as Alzheimer’s [38,39]. Increasing cholinergic tone ie the levels of acetylcholine with the use of cholinesterase inhibitors such as donepezil has been used to address cognitive decline in mild to moderate Alzheimer’s disease. The Barnes maze has been used to assess learning and memory in rodents [35]. It has several advantages over the more commonly used water maze in that it is less stressful for mice. It is an extensively used tool in behavioural neuroscience to investigate spatial learning and memory. Scopolamine which causes impairments in Barnes maze testing can be reversed by increasing cholinergic tone by the administration of a cholinesterase inhibitor such as donepezil [35]. Memory can be divided to short-term or long term memory where short term memory refers to holding information in conscious awareness for a duration of seconds whereas long term memory holds a larger amount of information for a longer period of time [40]. Working memory is a subset of short-term memory, required to perform certain mental operations during retention [41]. The Barnes radial maze has been used to assess learning and memory including working memory [42] seen in the animals of this study when locating the correct escape hole. Working memory errors are scored in this task as revisits to “incorrect” holes which subjects have already investigated within a probe trial. Cholinergic (acetylcholine) systems influence long term and working memory [43] as seen in the higher dose of *P. minus* and *G. biloba* group where the animals spent a longer duration in the target segment during probe trial as an indicator that the target segment location is remembered. The mice in lower doses of *P. minus*, the donepezil and scopolamine group demonstrated a shorter period within the target segment suggesting poorer memory for the target segment location that it was exposed to initially during the probe trial. The *in vitro* data from the present study, demonstrates that the extract has measurable anticholinesterase activity (68%), hence it may be that the extract induced increases in cholinergic tone, additionally providing an explanation for the attenuation of scopolamine induced deficits.

Scopolamine memory impairments have also been associated with brain oxidative stress [44] and scopolamine has been shown to trigger the induction of reactive oxygen species and to cause free radical injuries [45]. Herbal extracts with high antioxidant activity have been reported to scavenge free radicals and prevent scopolamine induced lipid peroxidation [26]. *P. minus* has been reported to possess up to 98.3% of lipid peroxidation inhibitory activities [2]. The antioxidant property of *P. minus*[8] may have contributed to the reactive oxygen species scavenging activity thus improving cognition and protecting against cognition decline [15]. It has been suggested that some of these antiamnesic effects are a direct result from antioxidant activity.

In considering human dosage, safety has been demonstrated in the acute toxicity test where oral administration of 2000 mg/kg of the standardised *P. minus* extract used in this study produced neither mortality nor changes in behavior or any other physiological activities [46]. The same paper reported in subacute 28-days for the dose of 1000 mg/kg, the no-observed-adverse-effect-level (NOAEL) of the extract was found to be more than 1000 mg/kg body weight in Wistar rats. Blood chemistry analysis including total protein, albumin, globulin, alanine transaminase (ALT), aspartate transaminase (AST), alkaline phosphatase (ALP), glucose, creatinine, urea nitrogen, total billirrubin, calcium, phosphorous, cholesterol, triglycerides, sodium and potassium and haemotological analysia in animals of both sexes, showed no significant changes at 1000 mg/kg.

In a recent randomized, double-blind, placebo-controlled crossover study of a propriety herbal blend (SuperUlam) containing 150 mg of *P. minus* water extract as one of its ingredient, natural ingredients in brain health of individuals aged 35–65 years of age was evaluated [47]. There was an improvement in cognitive function based on computer assisted testing, demonstrated by a significant improvement from baseline in executive functioning, cognitive flexibility, reaction time, and working memory in subjects on the propriety herbal blend. There was a significant decrease in tension, depression, and anger measured by the Profile of Mood Scores (POMS) in subjects that consumed the blend when compared to placebo. It is possible that the improvement in cognition was a result of *P. minus* which was one of its major ingredient.

## Conclusion

The present study confirms that water extract of *P. minus* has antioxidant activity with a high ORAC value of 16,964 μmole TE/gram and was able to reduce oxidative stress in a dose dependent manner. Higher dose of *P minus* (100 mg/kg) was able to attenuate scopolamine induced deficit in cognition *in vivo* by a reduction of total path length travelled and total errors prior to finding escape hole and increased duration in target segment during probe trial, indicating improved memory. These properties suggest that further investigations into the therapeutic potential of this extract for cognition could be a fruitful endeavour.

## Competing interests

The authors declare that Annie George and Wong Hoi Jean are employees of Biotropics Malaysia Bhd. Biotropics Malaysia Bhd funded this study and the article processing fee. Chee Perng Ng, Matthew O’Callaghan and Gitte S. Jensen declare they have no conflict of interest. The findings of the study have been applied for patent by Biotropics Malaysia Bhd.

## Authors’ contributions

AG was responsible for the conception of the study and participated in the *in vivo* design and worked on the draft of manuscript. CPN and MOC designed, conducted and interpreted the research outcome of the *in vivo* study, performed the statistical analysis and worked on the draft of manuscript. GSJ performed the cell based antioxidant assay and made contribution to the revision of the draft manuscript. HJW worked on the standardisation of the herbal extract used in this study. All authors read and approved the final manuscript.

## Pre-publication history

The pre-publication history for this paper can be accessed here:

http://www.biomedcentral.com/1472-6882/14/161/prepub
